# *In vitro *effects of 2-methoxyestradiol-bis-sulphamate on reactive oxygen species and possible apoptosis induction in a breast adenocarcinoma cell line

**DOI:** 10.1186/1475-2867-11-43

**Published:** 2011-12-12

**Authors:** Michelle H Visagie, Anna M Joubert

**Affiliations:** 1 Department of Physiology, University of Pretoria, P.O. Box 2034, Pretoria, 0001, South Africa

**Keywords:** 2-methoxyestradiol-bis-sulphamate, cell cycle progression, reactive oxygen species, mitochondrial integrity, apoptosis, autophagy

## Abstract

**Background:**

In the search for anticancer agents, a promising 17-β-estradiol metabolite, 2-methoxyestradiol (2ME2) was found that exerts antiproliferative *in vitro *and *in vivo *activity. Since 2ME2 has limited biological accessibility and rapid metabolic degradation, the purpose of this study was to investigate the *in vitro *influence exerted by an analogue of 2ME2 namely 2-methoxyestradiol-bis-sulphamate (2MEBM) in a breast adenocarcinoma cell line (MCF-7).

**Methods:**

This was conducted by investigating 2MEBM's *in vitro *influence on cell cycle progression, mitochondrial membrane potential and possible production of reactive oxygen species (ROS) generation. *In vitro *effects of 2MEBM on cell cycle progression was demonstrated by means of flow cytometry using propidium iodide. Hydrogen peroxide and superoxide production was investigated using 2,7-dichlorofluorescein diacetate and hydroethidine, respectively. The probable reduction in the mitochondrial membrane potential was demonstrated using a MitoCapture™ kit.

**Results:**

Cell cycle progression revealed the presence of a sub-G_1 _apoptotic peak. Reduction of mitochondrial membrane potential after exposure to 2MEBM was demonstrated and an increase in ROS production was also observed.

**Conclusion:**

This study verified that 2MEBM exposure resulted in apoptosis induction, increased ROS production and reduced mitochondrial membrane potential in a tumorigenic breast epithelial cell line. Data obtained from this project contributes to the unravelling of the *in vitro *signal transduction of 2MEBM in tumorigenic cell lines.

## Introduction

2-Methoxyestradiol (2ME2), the major derivative of 2-hydroxyestrogens is an endogenous inhibitor of breast cancer development [1]. 2ME2 exerts its antiproliferative, antiangiogenic and anticancer *in vitro *and *in vivo *activity in an estrogen receptor independent mode [1-3]. Antiproliferative effects were observed in the estrogen receptor positive human breast adenocarcinoma cell line (MCF-7), highly tumorigenic melanoma cell line (MDA-MB-435) and the human cervical adenocarcinoma cell line (HeLa). In addition, 2ME2 also inhibited *in vivo *growth of xenografts generated from MDA-MB-435 melanoma cells, murine sarcoma cells (Meth A sarcomas), murine melanoma cells (B16 sarcomas) and the human multiple myeloma KAS-6/1 cell line [3]. 2ME2 (patented as Panzem^®^) is currently undergoing phase II clinical trials as an anticancer drug for multiple myeloma [2], ovarian cancer [4], glioblastoma multiforme [5], breast- and prostate cancer [6]. However, high doses are needed (up to 6 g/day), since 2ME2 has limited biological accessibility due to the rapid inactivation of the hydroxyl groups at positions C3/C17 by conjugation and oxidation [7].

Low oral bioavailability and fast metabolic degradation of 2ME2 resulted in the chemical production of promising 2ME2 analogues. One of these analogues is 2-methoxyestradiol-bis-sulphamate (2MEBM), a bis-sulphamoylated derivative of 2ME2 that possesses antiproliferative and antitumor activity with enhanced bioavailability [8-11]. *In vitro *human umbilical vein endothelial cells (HUVEC) growth-inhibition assay studies indicated that 2MEBM is 60-fold more potent than 2ME2 as antiangioangenic agent [3] and tenfold more potent than 2ME2 as an antiproliferative agent [3,12]. Increased potency is related to the additional sulphamate group added to the precursor 2ME2 molecule [3,12].

Antiproliferative effects of 2MEBM have been reported in tumour growth in nude mice bearing xenografts derived from MDA-MB-435 cells, estrogen receptor positive human breast adenocarcinoma wild type cell line (MCF-7_wt_), mitoxantrone resistant breast adenocarcinoma cell line (MCF-7 MR), drug resistant human adenocarcinoma cell line (MCF-7 DOX40), prostate cancer cell line (LNACaP) and human umbilical vein endothelial cells (HUVEC) [3,13-15]. 2MEBM induces a G_2_/M arrest with reversible morphology change in dermal fibroblasts from the elongated fibroblast morphology to a cobblestone, morphology with rounding of cells, while the same treatment in human umbilical vein endothelial cells did not induce morphological changes or a G_2_/M arrest, indicating that the effects of 2MEBM on growth is cell line dependent [16]. A G_2_/M arrest followed by the induction of apoptosis was however observed in cell lines including tumorigenic estrogen receptor positive breast carcinoma cell line (MCF-7), drug resistant human adenocarcinoma cell line (MCF-7 DOX40), mitoxantrone resistant breast adenocarcinoma cell line (MCF-7 MR) and highly tumorigenic estrogen receptor negative breast carcinoma cell line MDA-MB-231 [8,17].

Since several questions remain regarding the *in vitro *influence of 2MEBM, this study aimed to investigate whether 2MEBM alters cell cycle progression and mitochondrial membrane integrity. The influence on reactive oxygen species production in the tumorigenic breast epithelial MCF-7 cell line was also studied.

## Materials and methods

### Materials

#### Cell line

MCF-7 is a tumorigenic adherent breast epithelial cell line derived from metastatic sites in adenocarcinoma. MCF-7 cells are able to process estradiol via cytoplasmic estrogen receptors and are capable of forming domes. MCF-7 cells were supplied by Highveld Biological (Pty) Ltd. (Sandringham, South Africa).

#### Reagents

All required reagents of cell culture analytical grade were purchased from Sigma (St. Louis, United States of America) unless otherwise specified. Heat-inactivated fetal calf serum (FCS), sterile cell culture flasks and plates were purchased from Sterilab Services (Kempton Park, Johannesburg, South Africa). Penicillin, streptomycin and fungizone were obtained from Highveld Biological (Pty) Ltd. (Sandringham, South Africa). 2,7-Dichlorofluorescein diacetate and hydroethidine was acquired from Sigma (St. Louis, United States of America). The MitoCapture ™ Mitochondrial apoptosis detection kit was purchased from BIOCOM biotech (Pty) Ltd. (Clubview, South Africa). Since 2MEBM is not commercially available, it was synthesized by Professor Vleggaar from the Department of Chemistry (University of Pretoria, Pretoria, South Africa). The fluorescence activated cell sorting (FACS) FC500 System flow cytometer equipped with an air-cooled argon laser excited at 488 nm was supplied by Beckman Coulter South Africa (Pty) Ltd. (Pretoria, South Africa).

#### Cell culture

Cells were grown and maintained in 25 cm^2 ^tissue culture flasks in a humidified atmosphere at 37°C and 5% CO_2_. MCF-7 cells were cultured in Dulbecco's minimum essential medium eagle (DMEM) and supplemented with 10% heat-inactivated FCS (56°C, 30 min), 100 U/ml penicillin G, 100 μg/ml streptomycin and fungizone (250 μg/l).

#### General cell culture procedures for experiments

A stock solution of 2 × 10^-3^M 2MEBM dissolved in dimethyl sulphoxide (DMSO) was prepared and diluted with medium to the desired concentrations prior to exposure of the cells. Medium of control cells was supplemented with an equal volume of DMSO (vehicle control cells). The DMSO content of the final dilutions never exceeded 0.05% (v/v). Experiments were conducted in 25 cm^2 ^cell culture flasks with exponentially growing MCF-7 cells seeded at 1 × 10^6 ^cells per 25 cm^2 ^flask to a final volume of 5 ml of maintenance medium. After a 24 h incubation period at 37°C to allow for cell adherence, medium was discarded and cells were exposed to 2MEBM. Previous data obtained in our laboratory by means of crystal violet staining revealed that the 50% growth inhibitory concentration (IC_50_) of 2MEBM on the tumorigenic MCF-7 cell line was found to be after 48 h of exposure and a concentration a 0.4 μM [18,19]. All subsequent experiments were thus performed at 48 h exposure period with a 0.4 μM 2MEBM on MCF-7 cells. Cells exposed to actinomycin D with a concentration of 0.1 μg/ml in growth medium for 48 h served as a positive control for apoptosis.

### Methods

#### Cell cycle progression

Cell cycle distribution, G_2_/M block and the detection of a sub-G_1 _apoptotic peak were analysed by flow cytometry using propidium iodide DNA staining. After 48 h of exposure to 0.4 μM 2MEBM, cells were trypsinized and resuspended in 1 ml growth medium. 1 × 10^6 ^cells were centrifuged for 5 min at 300 xg. The pellet resuspended twice in ice-cold phosphate buffer solution (PBS). The supernatant was discarded and the cells were resuspended in 200 μl of ice-cold PBS containing 0.1% FCS. Ice-cold 70% ethanol (4 ml) was added in a drop wise manner and cells were stored at 4°C for 24 h. After 24 h, cells were pelleted by centrifugation for 5 min. The supernatant was removed and cells were resuspended in 1 ml of PBS containing propidium iodide (40 μg/ml) and incubated at 37°C, 5% CO_2 _for 45 min. Subsequently, cells were analysed by means of FACS FC500 System flow cytometer (Beckman Coulter South Africa (Pty) Ltd) equipped with an air-cooled argon laser excited at 488 nm. Cell cycle distributions were calculated from at least 10 000 cells and were analyzed means of cyflogic version 1.2.1 software (Pertu Therho, Turko, Finland) by assigning relative DNA content per cell to sub-G_1_, G_1_, S and G_2_/M fractions.

#### Mitochondrial membrane potential

Mitochondrial integrity was investigated by means of a unique cationic dye, 5,5',6,6'-tetrachloro-1,1',3,3'-tetraethylbenzimidazolylcarbocyanine iodide. The MitoCapture ™ mitochondrial kit provides quantitive apoptosis information. Reduction of the mitochondrial membrane potential is an early feature during apoptosis due to the loss of the electrochemical gradient across the mitochondrial membrane [20]. Actinomycin D will be utilised as a positive control for reductions in the mitochondrial membrane [21]. After 48 h of exposure to 0.4 μM 2MEBM, cells were trypsinized and centrifuged at 13 000 × g. Cells were resuspended in 1 ml of diluted MitoCapture solution (1 μl MitoCapture: 1 ml pre-warmed incubation buffer), incubated at a humidified atmosphere (37°C, 5% CO_2_) for 20 min and subsequently centrifuged at 500 × g. After the supernatant was discarded, cells were resuspended in 1 ml of prewarmed incubation buffer (37°C). Cells were analyzed immediately after following the above-mentioned step using fluorescence activated cell sorting (FACS) FC500 System flow cytometer (Beckman Coulter South Africa (Pty) Ltd). Apoptotic cells were detected in the FITC channel (usually FL1) showing diffused green fluorescence. Data from at least 10 000 cells were analyzed by means of cyflogic version 1.2.1 software (Pertu Therho, Turko, Finland).

#### Hydrogen peroxide measurement

Hydrogen peroxide (H_2_O_2_) generation was assessed using 2,7-dichlorofluorescein diacetate (DCFDA), a non-fluorescent probe, which, upon oxidation by ROS and peroxides is converted to the highly fluorescent derivative 2,7-dichlorofluorescein (DCF). After 48 h of treatment, cells were trypsinized and 1 × 10^6 ^cells were resuspended in 1 ml PBS. Cells were incubated with 20 μM DCFDA for 25 min at 37^°^C. Hydrogen peroxide (20 μM) was added 5 min prior to measurement as a positive control for DCF formation. DCF (FL1) fluorescence was measured with a FACS FC500 System flow cytometer (Beckman Coulter South Africa (Pty) Ltd.) equipped with an air-cooled argon laser excited at 488 nm. Data analysis from at least 10 000 cells were conducted using cyflogic version 1.2.1 software (Pertu Therho, Turko, Finland).

#### Superoxide measurement

Superoxide generation was assessed using hydroethidine (HE). HE is oxidized by superoxide and not by hydroxyl radicals, singlet O_2_, H_2_O_2 _or nitrogen radicals, to a red fluorescent compound. After 48 h of treatment, cells were trypsinized and 1 × 10^6 ^cells were resuspended in 1 ml PBS. Cells were incubated with 10 μM HE for 15 min at 37^°^C. The HE fluorescent product fluorescence (FL2) was measured using a FACS FC500 System flow cytometer (Beckman Coulter South Africa (Pty) Ltd.) equipped with an air-cooled argon laser excited at 488 nm. The information generated from at least 10 000 cells were analyzed by means of cyflogic version 1.2.1 software (Pertu Therho, Turko, Finland).

#### Statistics

Measurement of FITC-, HE- and DCF-derived fluorescence was expressed as a ratio of the value measured for the 2MEBM-treated cells compared to vehicle-treated exposed cells (mean relative fluorescence). Flow cytometry analysis involved data from at least 10 000 events that was repeated thrice where after a representative figure was chosen for each experiment.

## Results

### Cell cycle progression

**Figure 1:**
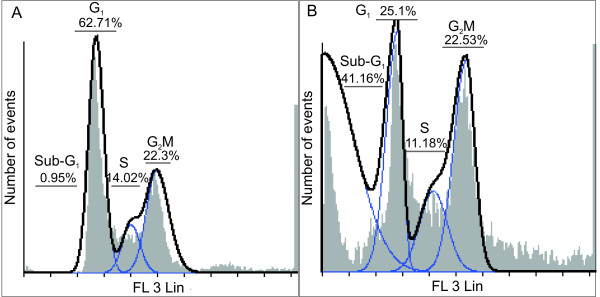
**Cell cycle histograms (FL3 Log representing propidium iodide detected by flow cytometry) revealed a statistical significant sub-G_1 _apoptotic increase in 0.4 μM 2ME2BM-treated cells (B) when compared to vehicle-treated cells (A)**. However, no G_2_/M block was observed in the 0.4 μM 2ME2BM-treated cells. A decrease in cells present in G_1 _was found in the 0.4 μM 2ME2BM-treated cells when compared to the vehicle-treated cells.

DNA content analyses by means of flow cytometry showed no increase of cells occupying the G_2_/M phase in 2MEBM-treated cells compared to vehicle-treated cells. However, a statistically significant increase was observed in the sub-G_1 _population when compared to the vehicle-treated cells. Furthermore, a reduction of 2MEBM-treated cells in the G_1_-phase was found when compared to the vehicle-treated control (Figure 1A and 1B).

### Mitochondrial integrity

**Figure 2:**
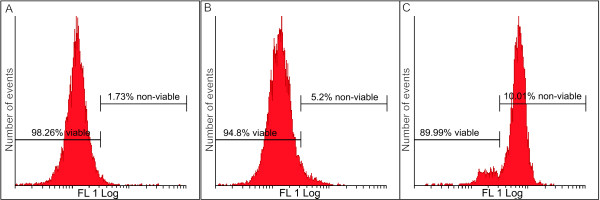
**Mitochondrial membrane potential was investigated by means of flow cytometry using the,5',6,6'-tetrachloro-1,1',3,3'- tetraethylbenzimidazolylcarbocyanine iodide dye (FL1 Log represented the dye detected by the flow cytometry)**. A reduction in mitochondrial membrane potential was observed in the 2ME2BM-treated cells (B) when compared to the vehicle-treated cells (A). Figure C depicts MCF-7 cells exposed to actinomycin D which was used as the positive control for the reduction of the mitochondrial membrane potential.

Flow cytometry analyses revealed a statistically insignificant reduction in the mitochondrial membrane potential in the 2MEBM-treated cells when compared to vehicle-treated cells and actinomycin D-treated cells (Figure 2A, B and 2C). The latter is an indicator of apoptosis due to the loss of the electrochemical gradient across the mitochondrial membrane.

### Hydrogen peroxide

**Figure 3:**
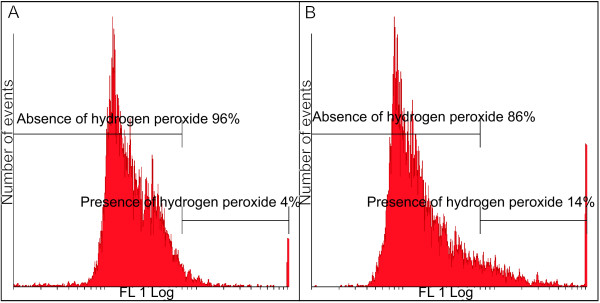
**Hydrogen peroxide measurement (FL1 Log representing the hydrogen peroxide detected by flow cytometry) in 2ME2BM-treated cells demonstrated a statistically insignificant increase to 14% when compared to the vehicle-treated cells (4%)**.

In order to investigate the hydrogen peroxide production in 2MEBM-treated cells compared to vehicle-treated cells, flow cytometry using DCFDA was employed. A statistically insignificant increase in hydrogen peroxide was detected in the 2MEBM-treated cells when compared to the vehicle-treated cells (Figure 3A and 3B).

### Superoxide measurement

**Figure 4:**
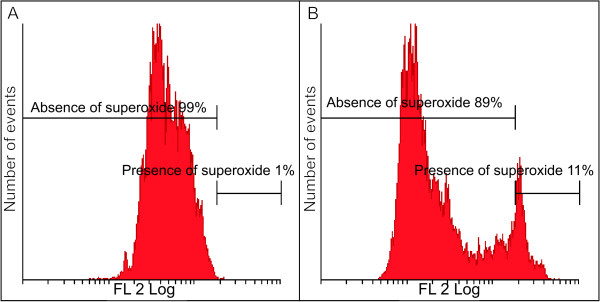
**Superoxide production detection (FL2 Log representing the superoxide detected by flow cytometry) revealed an increase of 10% in the 2ME2BM-treated cells when compared to the vehicle-treated cells**.

Flow cytometry utilizing HE demonstrated the influence of 2MEBM on superoxide production in MCF-7 cells. The latter was increased in a statistically insignificant manner in 2MEBM-treated cells when compared to the vehicle-treated cells (Figure 4A and 4B).

## Discussion

As previously stated, 2MEBM holds therapeutic potential as an anticancer agent, since it exerts antiproliferative effects *in vitro *and inhibits tumour growth *in vivo *[14]. In this article the effects of 2MEBM on the MCF-7 cell line was investigated by demonstrating the influence of 2MEBM on cell cycle progression, membrane integrity and possible production of reactive oxygen species in order to suggests the types of cell death induced by 2MEBM.

Previous reports have indicated that 2MEBM induces a G_2_/M arrest followed by induction of apoptosis in cell lines including MCF-7, MCF-7 DOX40, MCF-7 MR and MDA-MB-231 [8,17]. In addition, 2MEBM caused a significant apoptotic sub-G_1 _peak after 48 h. Day, *et al *(2009) observed similar results where 24 h 2MEBM exposure resulted in G_2_/M arrest; however, 48 h exposure resulted in a sub-G_1 _peak accompanied with no changes in the cell cycle distribution in the human 2MEBM-resistant cell line (A2780.140) derived from the human ovarian carcinoma cell line (A2780) [13]. B-Cell Lymphoma-2 Family Members (BCL-2) phosphorylation has also been found to be induced by 2MEBM in MCF-7 cells and correlates with a G_2_/M arrest [22]. Foster, *et al *(2008) reported similar findings pertaining to the decrease of 2MEBM-treated cells observed in the G_1_-phase in this study [8,22,23]. Induction of apoptosis by 2MEBM in breast adenocarcinoma CAL51 cell line was demonstrated by Wood, *et al *(2004) [24]. In this study 2MEBM caused a reduction in the mitochondrial membrane potential, confirming the induction of apoptosis. The mitochondrial membrane potential depolarization is an early event in the intrinsic (mitochondrial) apoptotic pathway [20,25,26]. Our study suggests that 2-methoxyestradiol-bis-sulphamate (2MEBM) influences the mitochondrial membrane potential minimally, thus the involvement of the intrinsic pathway to a lesser extent. Furthermore, exposure to 2MEBM revealed a sub-G_1 _peak. This is confirmed by studies conducted in our laboratory where flow cytometry utilizing annexin V-FITC demonstrated that 2MEBM induced apoptosis in MCF-7 cells [18]. During apoptosis, the electrochemical mitochondrial membrane gradient collapses [27-30]. The latter precipitates cell death through either the release of molecules involved in apoptosis, or the loss of mitochondrial functions essential for cell survival [31].

This study demonstrates the novel finding that 2MEBM exposure in the MCF-7 cell line resulted in increased ROS production. However, the source and processing of ROS and the role they play in 2MEBM-induced growth inhibition and cell death remains to be elucidated as no reports has been published on this matter. It was previously reported that the precursor molecule, 2ME2, inhibits superoxide dismutase and increased ROS production in human leukaemia cells [32]. 2ME2 also induces ROS generation in cell lines including myeloid leukaemia U937 cells, myeloid leukaemia HL-60 cell line, human acute T cell leukaemia cells (Jurkat cells), HeLa cells and MCF-7 cells [32-35]. Azad, *et al *(2009) revealed that ROS can induce autophagy by means of Atg4, catalase and the mitochondrial electron transport chain leading to both cell survival and cell death and is a selective towards cancer treatment. ROS generation upregulates Beclin-1 expression by an unknown mechanism increasing the occurrence of autophagy and hydrogen peroxide is essential for starvation-induced autophagy and directly targets Atg4 for oxidation and inactivates Atg4 inducing autophagy [36]. In addition, upregulated mitochondrial matrix calcium leads to increased reactive oxygen species production results in a reduction of the mitochondrial membrane potential (permeability transition pore is triggered). The latter leads to cytochrome *c *release from the mitochondria and subsequent activation of caspases leading to apoptosis [37,38]. Ling, *et *al (2003) reported that upregulated reactive oxygen species was accompanied by a reduction in the mitochondrial membrane potential and subsequent apoptosis [39]. Thus, the increased ROS production accompanied with reduced mitochondrial potential found in this study suggests that both of the above-mentioned contribute to the apoptosis induced by 2MEBM. The novel finding regarding the *in vitro *influence of 2MEBM on either of these reactive oxygen species has not been reported as previously.

## Conclusion

In conclusion, the aims of this project namely, to investigate the effects of 2MEBM exposure in the MCF-7 cell line on cell cycle progression, reactive oxygen species production and the possible induction of cell death were achieved. 2MEBM exposure resulted in mitochondrial membrane potential reduction and increased ROS production and apoptosis. The data generated from this project contributes to the known scientific knowledge regarding the *in vitro *effects of 2MEBM, will possibly lead to new possible *in vitro *targets for cancer therapies. Future studies concerning the action mechanisms of 2MEBM is necessary to produce a better understanding regarding the treatment of cancer and may possibly contribute to the development and/or improvement of novel chemotherapeutic agents.

## Competing interests

The authors declare that they have no competing interests.

## Authors' contributions

MHV was involved in the project design, conducted the experiments and data analysis in this project and drafted the article. AMJ was involved in the planning of this project, project supervision, funding acquisition from various received grants, interpretation of data and revision of the manuscript. All authors have contributed and approved the final manuscript.
